# Efficacy and safety of apatinib combined with radiotherapy in the treatment of advanced pancreatic cancer: a meta-analysis

**DOI:** 10.1186/s12957-023-03055-0

**Published:** 2023-06-01

**Authors:** Yongli Ma, Jinghui Li, Liang Wen, Guosheng Zhang, Xueqing Yao

**Affiliations:** 1 Ganzhou Hospital of Guangdong Provincial People’s Hospital, Ganzhou Municipal Hospital, No. 49 Da Gong Street, Ganzhou, 341000 China; 2 Gannan Medical University, No. 1 Medical College Road, Ganzhou, 341000 China; 3 Department of Gastrointestinal Surgery, Department of General Surgery, Guangdong Provincial People’s Hospital (Guangdong Academy of Medical Sciences), Southern Medical University, No.106 Zhongshan 2Nd Road, Yuexiu District, Guangzhou, 510080 China

**Keywords:** Apatinib, Pancreatic cancer, Radiotherapy, Randomized control, Meta-analysis

## Abstract

**Objective:**

At present, pancreatic cancer (PC) has a high morbidity and mortality rate and a poor prognosis. The aim of this article was to study the efficacy and safety of apatinib combined with radiotherapy in the treatment of advanced PC.

**Methods:**

The PubMed, Cochrane Library, Embase, Wanfang, CNKI, VIP, and CBM databases were searched by computer to identify studies on the application of apatinib in patients with advanced PC. The patients in the included study were divided into an observation group (apatinib combined with radiotherapy) and a control group (radiotherapy only), and meta-analysis was performed for each outcome with Revman 5.4 software. This study was successfully registered on the PROSPERO website, and the registration number is CRD: 42,022,384,056 (available at https://www.crd.york.ac.uk/PROSPERO/display_record.php?RecordID=384056).

**Results:**

A total of 7 randomized controlled trials (RCTs) with 428 patients were included, including 215 in the observation group and 213 in the control group. Compared with the control group, the observation group showed a greater objective response rate [OR = 3.26, 95% CI (2.18, 4.87), *P* < 0.0001], disease control rate [OR = 5.04, 95% CI (3.12, 8.12), *P* < 0.0001], complete response rate [OR = 3.87, 95% CI (1.51, 9.88), *P* = 0.005], and partial response rate [OR = 2.43, 95% CI (1.63, 3.61), *P* < 0.001], The 1-year survival rate [OR = 2.39, 95% CI (1.15, 4.96), *P* < 0.05], 2-year survival rate [OR = 2.41, 95% CI (1.03, 5.61), *P* < 0.05], progression-free survival time [MD = 1.17, 95% CI (0.37, 1.96), *P* < 0.05], overall survival time [MD = 1.47, 95% CI (0.13, 2.80), *P* < 0.05], while the stability rate [OR = 1.14, 95% CI (0.72, 1.81), *P* = 0.58] and various complications were not significantly different between the two groups.

**Conclusion:**

Apatinib combined with radiotherapy was more effective than radiotherapy alone in the treatment of advanced pancreatic cancer (PC), and apatinib had acceptable safety. However, since our study was limited by the quantity and quality of the included studies, we look forward to more large-sample, multicentre, and high-quality RCTs in the future to verify the conclusions.

**Supplementary Information:**

The online version contains supplementary material available at 10.1186/s12957-023-03055-0.

## Introduction

In 2020, approximately 495,800 new patients with pancreatic cancer (PC) and 466,000 deaths from PC were reported worldwide [1]. PC is highly malignant, difficult to diagnose early, and usually advanced once detected, with approximately 80% of patients having lost the opportunity for surgical resection [2]. Systemic chemotherapy, including FOLFIRINOX (5-fluorouracil, leucovorin, irinotecan, and oxaliplatin) and gemcitabine, can prolong the median survival of patients with PC, but the treatment effect is still limited. The current low 5-year overall survival (OS) rate and multiple adverse reactions of these drugs indicate limited efficacy and safety [3]. At present, radiation therapy plays an important role in the treatment of PC. Some studies showed that standard extracorporeal radiotherapy combined with chemotherapy reduced the local progression rate of PC (32% vs. 46%, *P* = 0.03); however, the OS of PC patients did not significantly improve (HR 1.03; 95% CI 0.79–1.34, *P* = 0.83) [4]. The limitations of radiotherapy and chemotherapy, their side effects and tumor resistance make new approaches to PC treatment necessary.

Targeted therapy brings new hope to PC patients. Currently, existing clinical studies show the efficacy of apatinib in the treatment of advanced PC [5]. There are also basic studies showing that apatinib can inhibit the proliferation and migration of PC cells [6, 7]. The results of several clinical randomized controlled trials (RCTs) showed that the local control rate and tumor survival outcome of LAPC patients in the apatinib and SBRT treatment groups were significantly higher than those of patients in the SBRT treatment alone group (*P* < 0.05) [8–14]. However, there are differing results for adverse reactions: some studies suggested that the difference between the two groups was not statistically significant [7–10, 13]. There are also studies showing that the adverse effects in the apatinib and SBRT treatment group were significantly lower than those in the SBRT treatment alone group [14]. Another study proposed that the overall incidence of adverse effects in the apatinib and SBRT group was higher than that in the SBRT alone group [12]. The reason for the difference may be due to the small sample size.

The aim of this study was to perform a meta-analysis of published RCT studies comparing apatinib combined with radiotherapy and radiotherapy alone for advanced PC treatment, hoping to provide new insights and ideas and to benefit clinical decisions for the treatment of advanced PC.

## Materials and methods

### Study subjects and the exclusion criteria

This meta-analysis was registered in PROSPERO (registration number: CRD42022384056). The subjects included in the study were patients who were diagnosed with advanced unresectable PC. Objective data were collected from randomized controlled studies that were available for comparison. The interventions and groups were as follows: (1) the observation group: apatinib combined with radiotherapy, and (2) the control group: radiation therapy only. The exclusion criteria were (1) studies not published in Chinese and English; (2) expert consensuses, case reports, comments, animal experiments, reviews, nursing and other literature; and (3) duplication research publications.

### Outcomes

The outcomes were the effective rate, the clinical benefit rate, the complete response rate, the partial response rate, the stability rate, the progression rate, the 1-year survival rate, the 2-year survival rate, progression-free survival time, the overall survival period, leukopenia, proteinuria, nausea, vomiting, radiation-induced inflammation, liver and renal injury, hypertension, and thrombocytopenia (Table 1). The following were determined according to the WHO evaluation criteria: (1) the complete response (CR); (2) the partial response (PR); (3) stable disease (SD); and (4) progressive disease (PD). The objective response rate (ORR) and disease control rate (DCR) were calculated as follows: ORR = CR + PR and DCR = CR + PR + SD, respectively.

**Table 1:** Basic characteristics of included studies

Study	Year	Classification	Stage	Number	Age(years)		Intervention study		Period	Outcome measures
				*T/C*	*T*	*C*	*T*	*C*	*T/C*	
Chengming Wei [8]	2019	PC	IV	31/31	44.67 土 4.32	44.82土 4.41	Apatinib (500 mg, qd) + SBRT	SBRT	6 weeks	①②③④⑤⑥⑦⑧⑨⑩⑪⑫⑬⑮⑯
Jue Wang [9]	2021	PC	IV	30/30	57.20 土 5.04	56.61土 4.32	Apatinib (500 mg, qd) + IMRT	IMRT	4 weeks	①②③④⑤⑥⑪⑫⑬⑭⑰
Linjia Wang [10]	2020	PC	IV	33/32	56.61 ± 4.97	57.03 ± 5.05	Apatinib (500 mg, qd) + SBRT	SBRT	6 weeks	①②③④⑤⑥⑪⑫⑬⑮⑯
Pengpeng Xu [11]	2021	PC	IV	31/30	44.36 ± 4.14	44.67 ± 4.78	Apatinib (500 mg, qd) + SBRT	SBRT	6 weeks	①②③④⑤⑥⑪⑫⑬⑯
Yinghui Deng [12]	2022	PC	IV	30/30	59.50 ± 14.5	59.00 ± 16.00	Apatinib (250 mg, qd) + Palliative radiotherapy	Palliative radiotherapy	4 weeks	①②③④⑤⑥⑦⑧⑬⑭⑮⑰
Zhaowei An [13]	2020	PC	IV	30/30	57.31 ± 7.14	54.81 ± 5.82	Apatinib (500 mg, qd) + 3DCRT	3DCRT	4 weeks	①②③④⑤⑥⑨⑩⑬⑯
Zhidong Xue [14]	2020	PC	IV	30/30	58.35 ± 5.38	59.21 ± 5.42	Apatinib (250 mg-500 mg, qd) + Palliative radiotherapy	Palliative radiotherapy	5–7 weeks	%1 ②③④⑤⑥⑪⑫⑬

*T* observation group, *C* control group, *PC* pancreatic cancer; Outcome indicators: ①CR, complete remission; ②PR, partial remission; ③SD, stabilize; ④PD, progress; ⑤ *ORR* objective response rate, ⑥ *DCR* disease control rate, ⑦ *OS* overall survival, ⑧ *PFS* progression-free survival, ⑨ 1-year survival rate, ⑩2-year survival rate, ⑪ leucopenia, ⑫ thrombocytopenia, ⑬ nausea and vomiting, ⑭ albuminuria, ⑮ radiation-induced inflammation, ⑯ liver and kidney damage, ⑰ hypertension。*SBRT* stereotactic body radiotherapy, *IMRT* intensity modulated radiation therapy,*3DCRT* three-dimensional conformal radiation therapy

### Literature search strategy

Study retrieval, screening and classification were performed by two researchers. When disagreement occurred, a third investigator was asked to decide. The search strategy was as follows: the PubMed, Cochrane Library, Embase, CNKI, Vip, Wanfang, and CBM databases were searched until November 28, 2022, for RCTs of apatinib combined with radiotherapy for PC treatment using subject words combined with free words. To search all literature relevant to the purpose of this study, we used the following search terms: apatinib, Pancreatic Cancer, Randomized Controlled Trial, random, and RCT, Extracting Data Using Excel Tables.

### Quality evaluation of the included studies

The quality of the RCTs was evaluated by the Cochrane collaboration tool with the following domains: (1) random allocation; (2) allocation concealment; (3) blinding; (4) loss to follow-up; (5) selective reporting, and (6) other bias.

### Statistical method

The outcome data were processed with RevMan5.4 software, and the odds ratio (OR) was selected as the effect index for dichotomous variables. Using 95% confidence intervals (CIs), data heterogeneity was tested by the chi-square test, essentially no statistical heterogeneity was considered at *I*^*2*^ < 50% and *P* > 0.1, we use fixed effects model, if *I*^*2*^ > 50% or *P* < 0.1, and we use random effects model. For continuous variables, the mean difference (MD) was used, and a meta-analysis result of *P* < 0.05 indicated that the difference between the two groups was statistically significant. If the heterogeneity was significant, a subgroup analysis was performed to explore the source of heterogeneity, and sensitivity analysis was performed to judge whether the pooled results were robust. If there are less than 10 studies included, it is considered as publication bias.

## Results

### Literature screening process and results

**Fig. 1:**
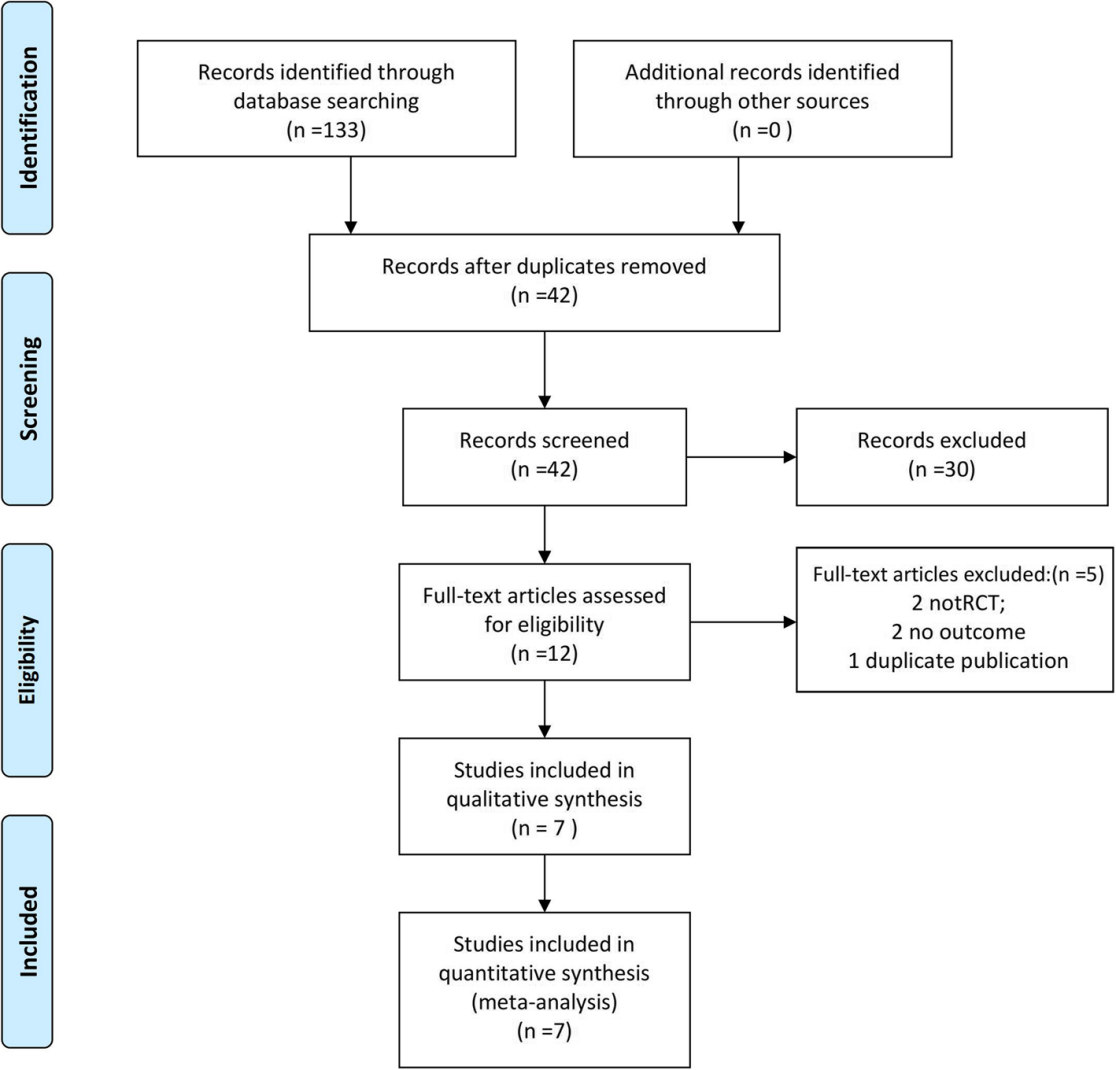
PRISMA flow diagram for study selection

The search yielded a total of articles. Seven RCTs [8–14] with a total of 428 patients were ultimately included, and the screening flow chart is shown in Fig. 1.

### Basic characteristics of the included studies

All included studies were from Chinese journals, and the authors were all Chinese nationals. Data on the first author, year of publication, outcome indicators, tumor classification, tumor stage, the number of samples, age, intervention measures, treatment course, and outcome measures were collected (Table 1).

### Results of the risk of bias assessment

**Fig. 2:**
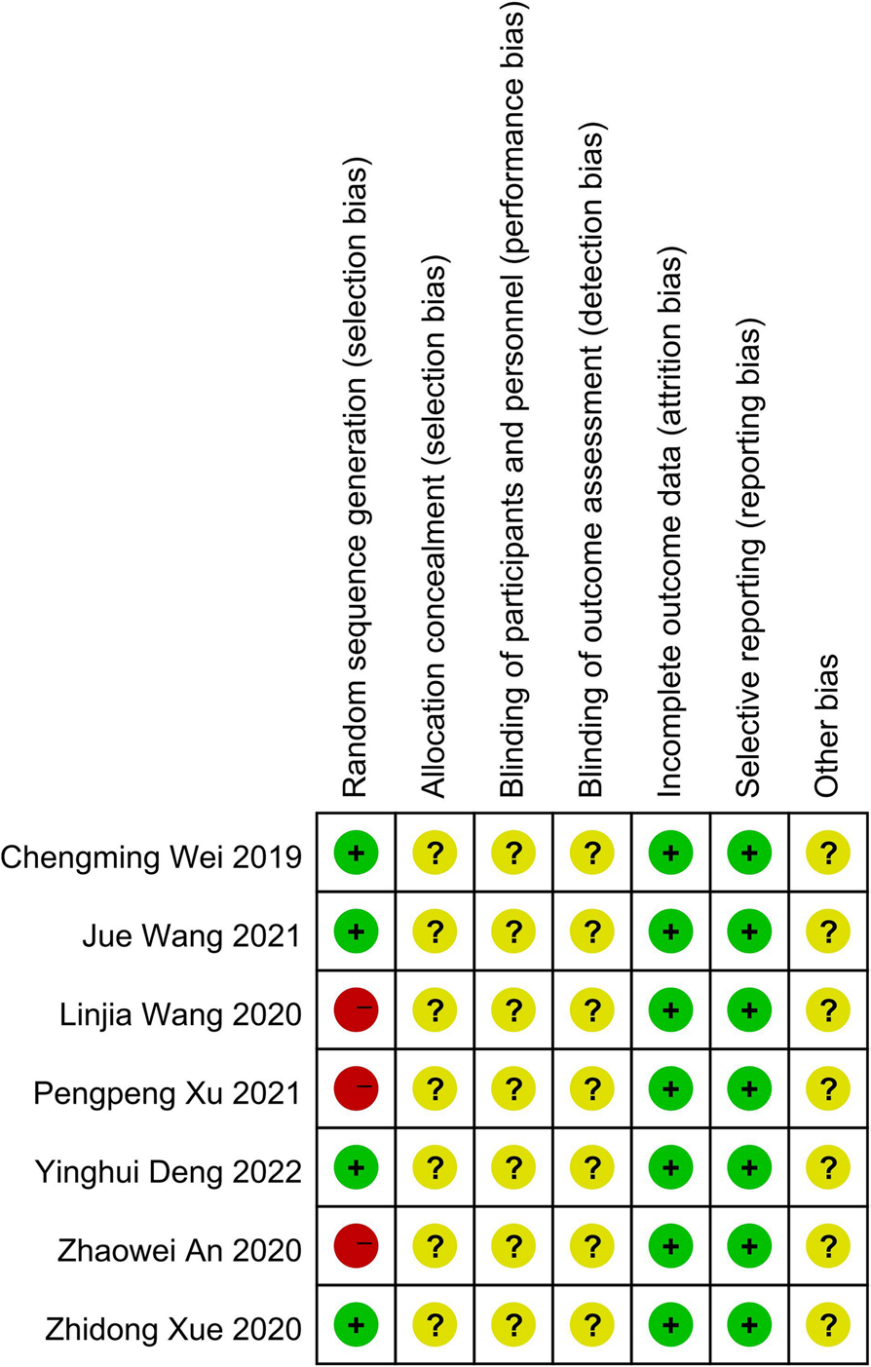
Summary of the risk of bias among the included studies

**Fig. 3:**
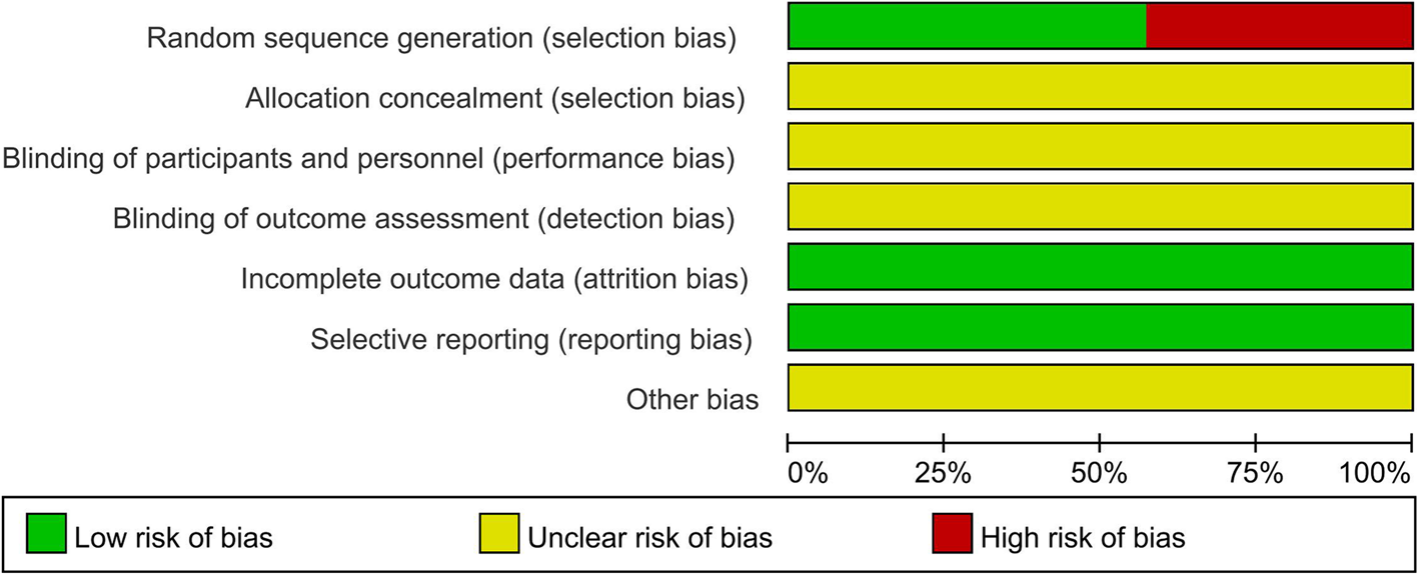
Risk of bias among the included studies

All included studies were RCTs, 4 included random numbers, and 3 did not specify how the random sequence was generated. None of the included studies mentioned concealment, blindness, withdrawal or loss to follow-up; selective reporting and other biases were not mentioned; and all studies mentioned that the baseline data of patient age and sex were comparable (*P* > 0.05). Methodological quality evaluation was performed using the Cochrane risk of bias assessment tool (Figs. 2 and 3).

### Meta-analysis results

#### Objective response rate and clinical benefit rate

**Fig. 4:**
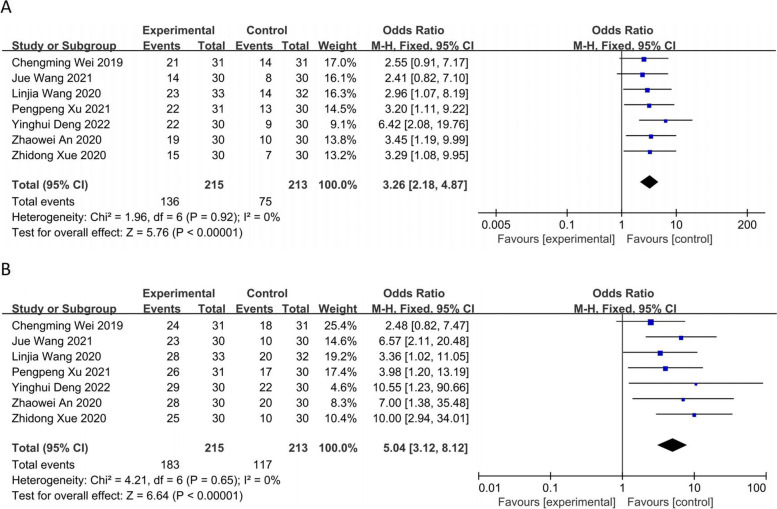
Forest plot: **A** objective response rate (ORR); **B** disease control rate (DCR)

The seven RCTs [8–14] including 428 patients reported the objective response rate (ORR) and a heterogeneity test was performed (*I*^*2*^ = 0%, *P* = 0.92) using a fixed-effects model. The observation group showed a greater ORR, and the difference between the two groups was statistically significant [OR = 3.26, 95% CI (2.18, 4.87), *P* < 0.00001] (Fig. 4A). Subgroup analysis suggested that different radiotherapy methods did not change the original conclusion (Supplemental Figure S1). The seven RCTs [8–14] patients reported the disease control rate (DCR) and a heterogeneity test was performed (*P* = 0.65; *I*^*2*^ = 0%) using a fixed effects model. The observation group showed a greater DCR, and the difference between the two groups was statistically significant [OR = 5.04, 95% CI (3.12, 8.12), *P* < 0.00001] (Fig. 4B). The subgroup analysis suggested that the original conclusion did not change despite the use of different radiotherapy methods (Supplemental Figure S2).

**Fig. 5:**
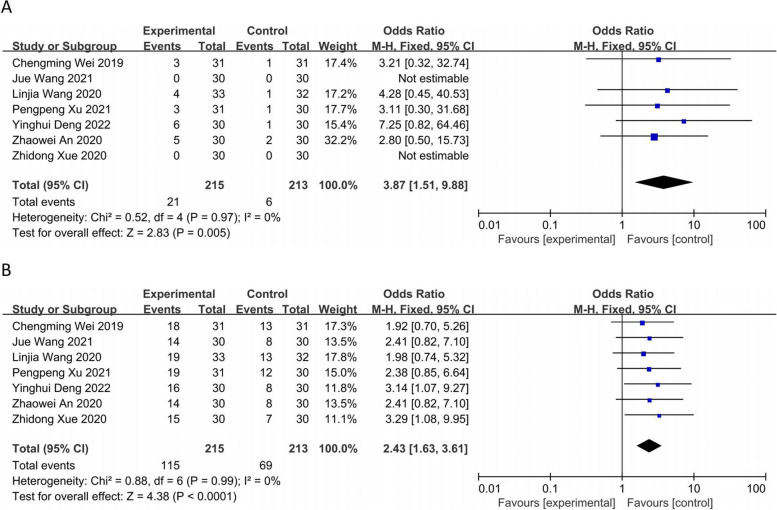
Forest plot: **A** complete response rate (CR); **B** partial response rate (PR)

The seven RCTs [8–14] including 428 patients reported the complete response rate (CR), and a heterogeneity test was performed (*P* = 0.97; *I*^*2*^ = 0%) using a fixed-effects model. The observation group showed greater PFS and the difference between the two groups was statistically significant [OR = 3.87, 95% CI (1.51, 9.88), *P* = 0.005] (Fig. 5A). The 7 RCTs [8, 14] patients reported partial response (PR) rates, and a heterogeneity test (*P* = 0.99; *I*^*2*^ = 0%) was performed using a fixed-effects model. The observation group showed greater PR, and the difference between the two groups was statistically significant [OR = 2.43, 95% CI (1.63, 3.61), *P* < 0.0001] (Fig. 5B).

**Fig. 6:**
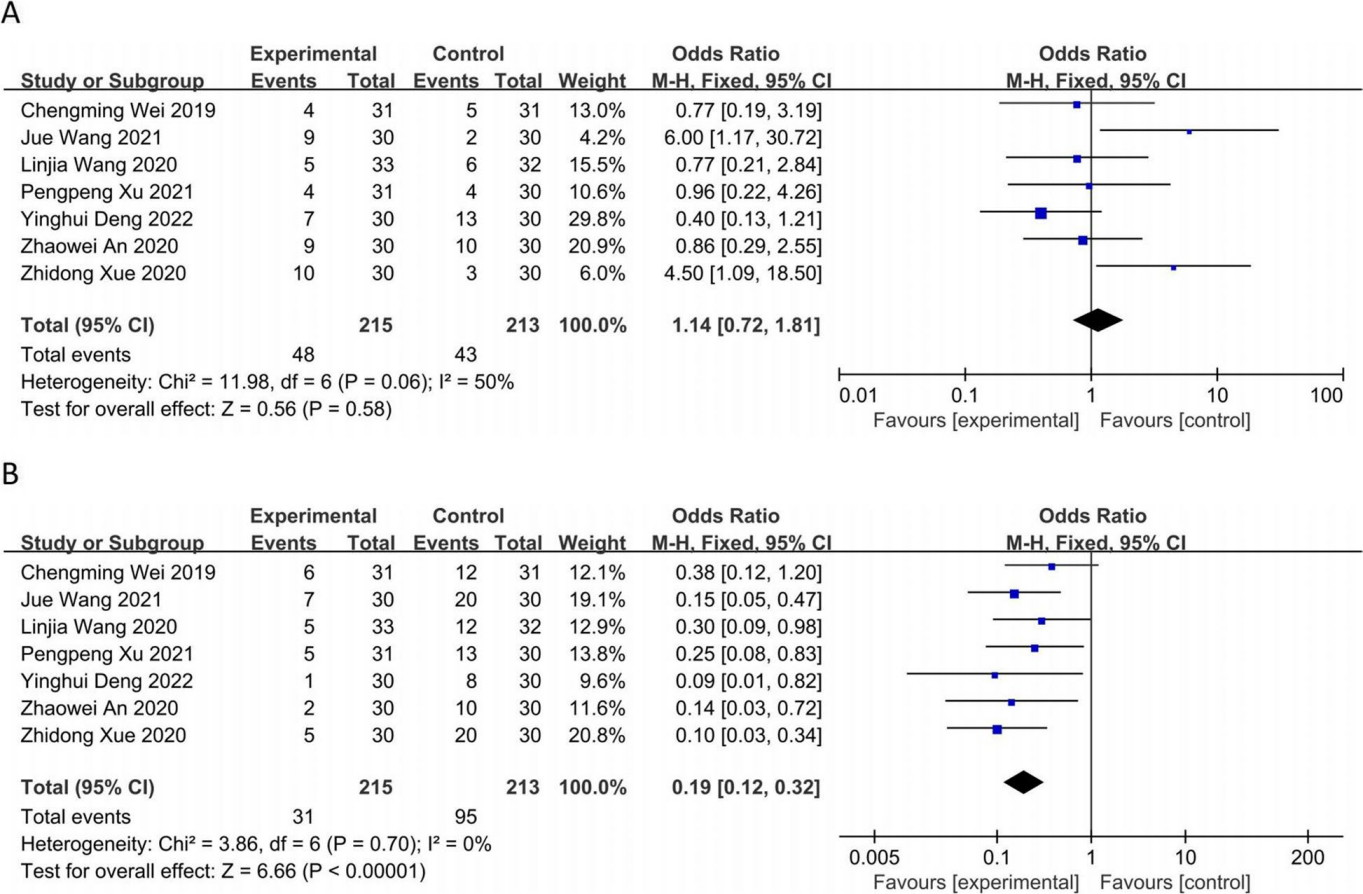
Forest plot: **A** treatment stability rate (SD); **B** treatment progression rate (PD)

The seven RCTs [8–14] including 428 patients reported the treatment stability rate (SD), performing a heterogeneity test (*P* = 0.06; *I*^*2*^ = 50%) using a fixed-effects model. The results showed no statistically significant difference between the two groups [OR = 1.14, 95% CI (0.72, 1.81), *P* = 0.58] (Fig. 6A). The seven RCTs [8–14] including reported the treatment progression rate (PD), performing a heterogeneity test (*P* = 0.70; *I*^*2*^ = 0%) using a fixed-effects model. The observation group showed a lower rate of PD, and the difference between the two groups was statistically significant [OR = 0.19, 95% CI (0.12, 0.32), *P* < 0.00001] (Fig. 6B).

**Fig. 7:**
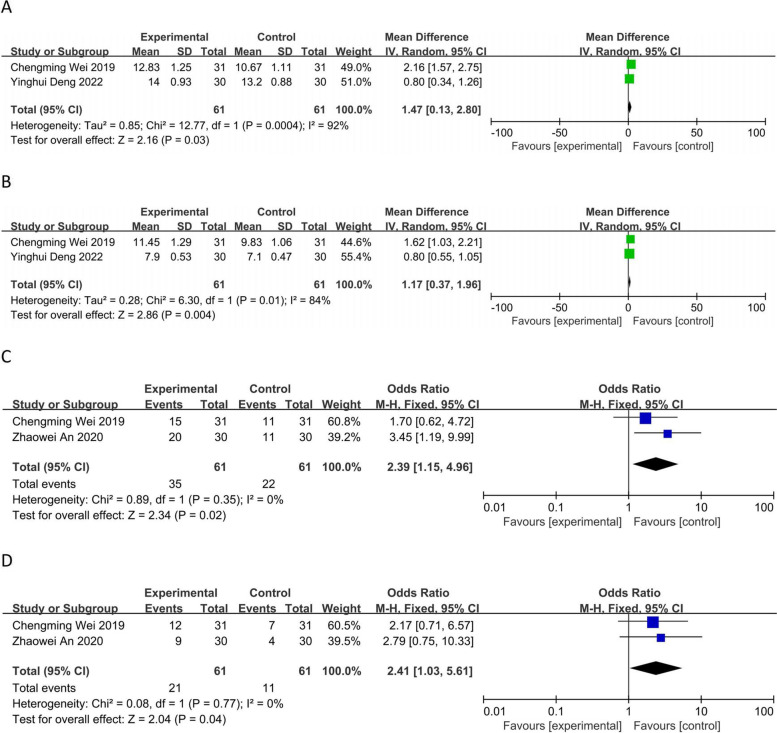
Forest plot:** A** Median OS; **B** progression-free survival (PFS); **C** 1-year OS rate; **D** 2-year OS rate

Two RCTs [8, 12] reported the median overall survival time (mOS), and a heterogeneity test (*P* = 0.0004; *I*^*2*^ = 92%) was performed using a random effects model. The observation group showed a greater mOS, and the difference between the two groups was statistically significant [MD = 1.47, 95% CI (0.13, 2.80), *P* < 0.05] (Fig. 7A). Two RCTs [8, 12] reported progression-free survival (PFS), and a heterogeneity test (*P* = 0.01; *I*^*2*^ = 84%) was performed using a random effects model.

The observation group showed a greater rate of PFS, and the difference between the two groups was statistically significant [MD = 1.17, 95% CI (0.37, 1.96), *P* < 0.05] (Fig. 7B). Two RCTs [8, 13] reported the 1-year survival rate, and a heterogeneity test was performed (*P* = 0.35; *I*^*2*^ = 0%) using a fixed-effects model. The observation group showed a higher 1-year OS rate, and the difference between the two groups was statistically significant [OR = 2.39, 95% CI (1.15, 4.96), *P* < 0.05] (Fig. 7C). Two RCTs [8, 13] reported the 2-year OS rate, and a heterogeneity test (*P* = 0.77;*I*^*2*^ = 0%) was performed using a fixed effects model. The observation group showed a higher 2-year survival rate, and the difference between the two groups was statistically significant [OR = 2.41, 95% CI (1.03, 5.61), *P* < 0.05] (Fig. 7D).

The meta-analysis showed no significant difference in complications between the two groups (*P* > 0.05) (Table 2). Complications included leukopenia, thrombocytopenia, nausea and vomiting, proteinuria, radiation-induced inflammation, hepatic and renal impairment, and hypertension. Among the complications, the incidences of radiation-induced inflammation, hypertension, proteinuria and other indicators were determined to be significantly heterogeneous using a random-effects model, while a fixed-effects model was used for the remaining outcome indicators.

**Table 2:** Meta-analysis results of apatinib combined with radiotherapy in the treatment of PC

			Heterogeneity test			Meta-analysis of the test results		
Prevalence of hepatic and renal damage	Number of studies	Sample capacity	*P*	*I*^*2*^	Effect model	MD/OR	95% CI	*P*
Radiation-induced inflammation	5	308	0.94	0%	Fixed	1.30	(0.68, 2.49)	0.43
Proteinuria	5	308	0.98	0%	Fixed	1.31	(0.62, 2.73)	0.48
Nausea and vomiting	7	428	0.64	0%	Fixed	1.01	(0.64, 1.58)	0.97
Thrombocytopenia	2	120	0.07	69%	Random	0.73	(0.04, 12.88)	0.83
Leukopenia	3	187	0.12	54%	Random	0.57	(0.13, 2.46)	0.45
Outcome indicators	4	248	0.95	0%	Fixed	1.09	(0.58, 2.04)	0.79
Hypertension	2	120	0.02	83%	Random	0.24	(0.00, 22.29)	0.54

### Publication bias analysis

Because the number of meta-analyses included was less than 10, this study is considered to have publication bias.

### Sensitivity analysis

The influence of each study on the risk estimate was investigated by removing studies one by one, which showed that the overall risk estimates were not obviously changed by any single study.

## Discussion

The PC 5-year OS rate was only 11% [15]. Surgery is also an effective means to promote long-term survival in patients with PC, but PC is so insidious and rapidly progressive that most patients are at an advanced stage at diagnosis and cannot undergo surgery [3]. Radiotherapy plays an important role in the treatment of PC. Stereotactic body radiotherapy (SBRT) is a new local radiotherapy technology that can form precise radiosurgical treatment by adjusting the grading and irradiation dose of radiation therapy and greatly improves the local control rate of PC [16–19]. Previous studies showed that SBRT combined with gemcitabine chemotherapy for patients with locally advanced pancreatic cancer (LAPC) showed better oncology outcomes: a median overall survival (mOS) of 13.9–16.7 months and a median progression-free survival (mPFS) of 6–10.2 months [20–22]. Compared to conventional fractionated radiotherapy, SBRT can reduce the incidence of adverse effects because of its precision [17, 23, 24]. A meta-analysis demonstrated an advantage of SBRT for LAPC patients in terms of OS and locoregional control (LRC) (1-year OS 51.6% and 1-year LRC 72.3%), with an incidence of serious adverse effects of less than 10% [25]. It has been shown that SBRT combined with monotherapy or multi-agent chemotherapy can increase the surgical opportunity in LAPC patients [26, 27]. However, relevant scholars have proposed that three-dimensional conformal radiation therapy (3-DCRT), intensity modulated radiation therapy (IMRT) and SBRT have similar local control outcomes for advanced PC [17, 28]. SBRT is dose-escalated to increase efficacy; however, many patients with LAPC cannot tolerate it. Therefore, some scholars choose to make changes to systemic therapy to increase efficacy, such as switching to capecitabine induction chemotherapy, SBRT combined with immunotherapy, and SBRT combined with targeted therapy [18, 19, 24]. Nevertheless, some studies indicated that the OS of patients treated with chemoradiotherapy was not prolonged compared with that in patients treated with chemotherapy alone [4]. The OS benefit brought to PC patients by the change in radiotherapy technology is not obvious and is controversial, so a new systemic treatment method is still worth active exploration.

Vascular endothelial growth factor (VEGF) plays a crucially important role in angiogenesis in PC treatment [19, 29]. For this target, relevant drugs, such as pazopanib, apatinib, nintedanib, and regorafenib, have been developed to block the expression of proangiogenic factors or to block their activity against tumors. Apatinib is also widely used in treating malignancies such as advanced gastric cancer, lung cancer, advanced colorectal cancer, and advanced liver cancer, showing significant efficacy and acceptable toxicity [30, 31]. Apatinib is a novel small-molecule tyrosine kinase inhibitor (TKI) that highly selectively competes for the associated binding sites of VEGFR-2 to inhibit its phosphorylation and the generation of vascular endothelial cells and tumor vessels. Apatinib can inhibit the expression of hypoxia-inducible factor-1α (HIF-1α), vascular endothelial growth factor and phosphoinositide 3-kinase (PI3K)/Akt/mTOR signalling pathway markers and promote apoptosis in PC cells [6, 7]. Related studies have noted that apatinib can reshape the tumor microenvironment and improve the expression level of tumor cell PD-L1 to inhibit the growth of tumors [32, 33]. The research also pointed out that the contrast of a single anti-PD-1 inhibitor combined with an antiangiogenic agent has a higher anti-tumor effect, which may be caused by the activation of T cells, strengthening the effect of immune function on cancer cells [34]. Apatinib was approved in China in 2014 for third-line treatment and above in patients with advanced gastric cancer or oesophageal-gastric junction adenocarcinoma [35]. To date, clinical treatment research on the use of apatinib in patients with gastric cancer, lung cancer, breast cancer, colorectal cancer, osteosarcoma and other malignant tumors has increased rapidly and has shown obvious therapeutic effects [36–38]. In view of the treatment of other cancers, scholars have applied apatinib in the treatment of advanced PC to research its efficacy and safety, but there is no consensus.

In our study, the results showed that apatinib plus radiotherapy had a superior objective response rate, disease control rate, complete response rate, and partial response rate and a lower progression rate, but there was no significant difference between the two groups in terms of stable disease. In terms of the objective response rate and disease control rate of two main outcome indicators, subgroup analysis showed that only the study of Wang Jue et al. showed no significant difference in the two groups [9]. Subgroup analysis of the rest of the included studies suggested that apatinib combined with radiotherapy had a better objective response rate and disease control rate, which shows that different methods of radiotherapy with effective and clinical benefit rates did not show obvious differences in the curative effect. The meta-analysis of two RCTs with related outcome measures showed that the apatinib combined with radiotherapy group had longer overall survival and progression-free survival times and higher 1-year and 2-year OS rates. In the five RCT meta-analyses of the incidence of leukopenia and thrombocytopenia, no significant difference was found; in the meta-analysis of the seven RCTs evaluating the incidence of nausea and vomiting, only two evaluated the incidence of proteinuria, radiation-induced inflammation, liver and kidney injury, hypertension, and proteinuria and summarized these data; no significant difference was found in the complication rate between the two groups. The results of this study were compared with those of a meta-analysis of lung cancer performed in 2021, which included 11 RCTs [39]. Regarding complications, there was no significant difference in drug-related adverse effects in hand-foot syndrome, gastrointestinal reactions, thrombocytopenia, anaemia, or leukocytopenia (*P* > 0.05). However, that study noted that the risk of hypertension was significantly higher in the apatinib group than in the control group (RR = 3.60, 95% CI 1.26–10.31, *P* < 0.05). The phenomenon of hand-foot syndrome and the higher incidence of hypertension were not found in this study. This may be limited by the sample size of this study. These aspects deserve further clinical exploration. In conclusion, apatinib is relatively safe and feasible for patients.

The advantages and limitations of this study are described as follows: To our knowledge, no meta-analysis has explored the efficacy and safety of apatinib combined with radiotherapy in the treatment of advanced PC. Our study has the following limitations: (1) the quantity and quality of the included studies was limited, and distribution concealment and blinding were not discussed in the studies. Publication bias, language bias, and implementation bias may exist; (2) factors such as the course of treatment, the chemoradiation regimen, and patient conditions were not consistently analysed in the included studies, which may affect the results,This may lead to heterogeneity; (3) some outcome measures (such as progression-free survival time, the 1-year survival rate, and the 2-year survival rate) were included in only two studies, and the reliability of the results needs to be strengthened; (4) there was a lack of multicentre studies; and (5) this study included only the Chinese population, and the conclusions may not be applicable in other populations. However, some studies [40, 41] from countries outside China have pointed out that other tyrosine kinase inhibitors, including loratinib and erlotinib, also play a role in the treatment of pancreatic cancer, which may provide new ideas for people around the world regarding pancreatic cancer treatment. We look forward to more research on apatinib in the treatment of pancreatic cancer around the world to benefit mankind.

In conclusion, apatinib combined with radiotherapy is more effective than radiotherapy alone for advanced PC treatment, which can improve the OS rate of patients, and apatinib has acceptable safety. In the future, more large-sample, multicentre, high-quality RCTs are needed to verify the conclusions of this paper.

## Supplementary Information

**Additional file 1:**  **Figure S1.** Meta-analysis of the objective response rate of apatinib combined with radiotherapy in the treatment of PC(different radiotherapy schemes). **Figure S2.** Meta-analysis of disease control rate of apatinib combined with radiotherapy in the treatment of PC (different radiotherapy schemes).

## Data Availability

All data generated or analyzed during this study are included in this published article.

## Abbreviations

PC: Pancreatic cancer
RCT: Randomized controlled trial
OS: Overall survival
SBRT: Stereotactic body radiotherapy
IMRT: Intensity modulated radiation therapy
3DCRT: Three-dimensional conformal radiation therapy
CR: Complete response
PR: Partial response
SD: Stable disease
PD: Progressive disease
ORR: Objective response rate
DCR: Disease control rate
LRC: Locoregional control
TKI: Tyrosine kinase inhibitor
HIF-1α: Hypoxia-inducible factor-1α
PI3K: Phosphoinositide 3-kinase
VEGF: Vascular endothelial growth factor
mPFS: Median progression-free survival
