# Supplementary material for: Efficacy and safety of apatinib combined with radiotherapy in the treatment of advanced pancreatic cancer: a meta-analysis
Source: World J Surg Oncol. 2023 Jun 1;21:165. doi: 10.1186/s12957-023-03055-0 (PMC10236655; doi:10.1186/s12957-023-03055-0)
Supplement: Supplementary file 1 — Additional file 1: Figure S1. Meta-analysis of the objective response rate of apatinib combined with radiotherapy in the treatment of PC(different radiotherapy schemes). Figure S2. Meta-analysis of disease control rate of apatinib combined with radiotherapy in the treatment of PC (different radiotherapy schemes). [file 12957_2023_3055_MOESM1_ESM.docx]

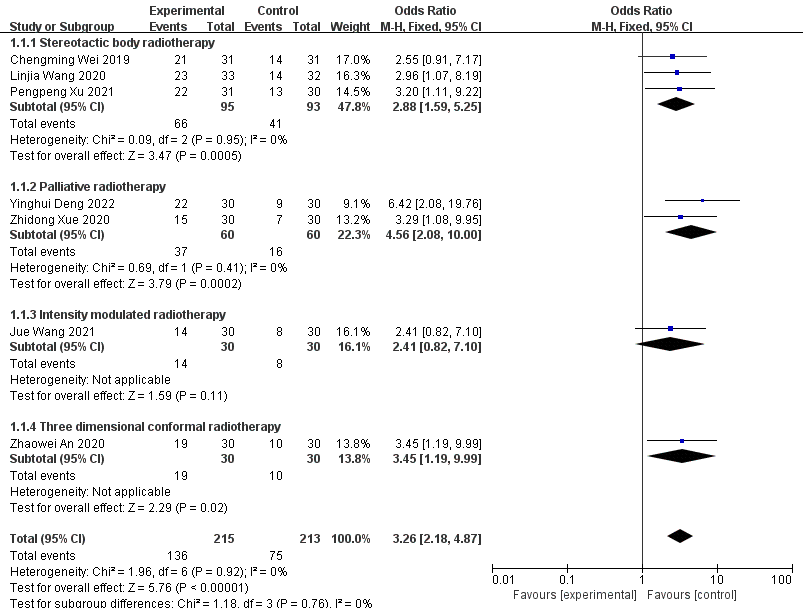


Figure A Meta analysis of the Objective response rate of apatinib combined with radiotherapy in the treatment of PC(different radiotherapy schemes)


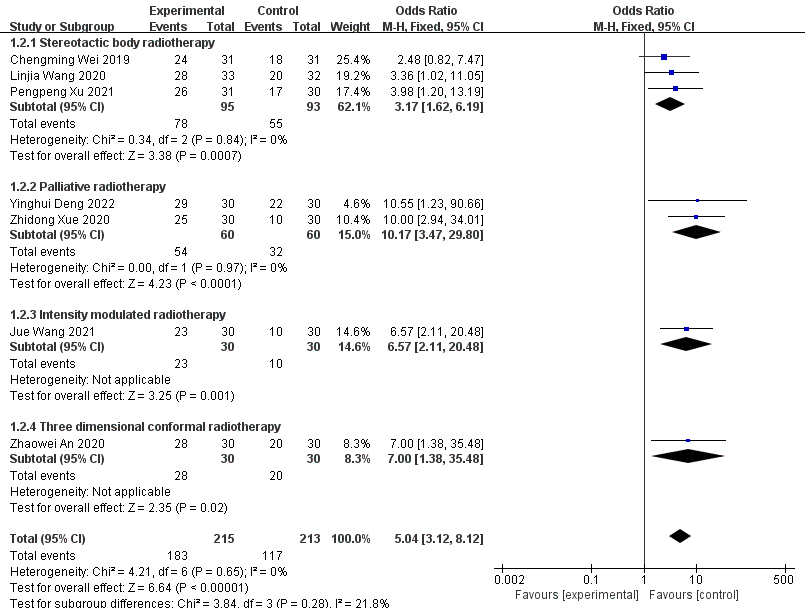


FigureB Meta analysis of disease control rate of apatinib combined with radiotherapy in the treatment of PC (different radiotherapy schemes)
